# Feline bocavirus-1 associated with outbreaks of hemorrhagic enteritis in household cats: potential first evidence of a pathological role, viral tropism and natural genetic recombination

**DOI:** 10.1038/s41598-019-52902-2

**Published:** 2019-11-08

**Authors:** Chutchai Piewbang, Tanit Kasantikul, Kidsadagon Pringproa, Somporn Techangamsuwan

**Affiliations:** 1 Department of Pathology, Faculty of Veterinary Science, Chulalongkorn University, Bangkok, 10330 Thailand; 2 Department of Preclinic and Applied Science, Faculty of Veterinary Science, Mahidol University, Nakhon Pathom, 73170 Thailand; 3 Department of Veterinary Biosciences and Veterinary Public Health, Faculty of Veterinary Medicine, Chiang Mai University, Chiang Mai, 50100 Thailand; 4 Diagnosis and Monitoring of Animal Pathogens Research Unit, Faculty of Veterinary Science, Chulalongkorn University, Bangkok, 10330 Thailand

**Keywords:** Infection, Pathogenesis, Infectious diseases

## Abstract

Feline bocavirus-1 (FBoV-1) was identified in cats from different households with hemorrhagic enteritis during outbreaks of an unusual clinical presentation of feline panleukopenia virus (FPLV) in Thailand. Use of polymerase chain reaction revealed the presence of the FBoV-1 DNA in several tissues, suggesting hematogenous viremia, with the viral nucleic acid, detected by *in situ* hybridization (ISH), was localized in intestinal cells and vascular endothelium of intestinal mucosa and serosa, and in necrosis areas primarily in various lymph nodes while FPLV-immunohistochemical analysis revealed viral localization only in cryptal cells, neurons, and limited to leukocytes in the mesenteric lymph node. Full-length coding genome analysis of the Thai FBoV-1 strains isolated from moribund cats revealed three distinct strains with a high between-strain genetic diversity, while genetic recombination in one of the three FBoV-1 strains within the NS1 gene. This is the first report identifying natural genetic recombination of the FBoV-1 and describing the pathology and viral tropism of FBoV-1 infection in cats. Although the role of FBoV-1 associated with systemic infection of these cats remained undetermined, a contributory role of enteric infection of FBoV-1 is possible. Synergistic effects of dual infection with FPLV and FBoV-1 are hypothesized, suggesting more likely severe clinical presentations.

## Introduction

Bocavirus (BoV), a linear single stranded DNA (ssDNA) virus with an approximately 5.5 kb length genome belongs to the genus *Bocaparvovirus* in the family *Parvoviridae*. The BoV genome possesses three main open reading frames, ORF1-3, encoding the non-structural protein NS1, capsid protein VP1/2 and nuclear phosphoprotein NP1, respectively^1^. The BoVs have emerged and caused diseases in various animals and humans, including canine BoVs (CBoVs), porcine BoVs (PBoV), bovine parvovirus, gorilla BoV, California sea lion BoV, rodent BoV, feline BoVs (FBoVs) and human BoVs (HBoVs), and so suggests a potentially wide host range of BoVs^1–3^. Many researches have indicated the association of BoVs with various clinical symptoms. Respiratory and intestinal diseases have been observed frequently in BoV-infected hosts^4^, but other uncommon clinical presentations have also been reported. These include encephalomyelitis in a PBoV-infected pig, necrotizing encephalitis in HBoV-infected humans and hepatitis in CBoV-infected dogs^5–7^. Likewise, previous studies have reported the detection of the BoV genome in liver, lymph node, feces and blood of infected hosts, suggesting that BoVs could cause systemic infections^2^.

The FBoVs, comprised of FBoV-1 to −3 and belong to *Carnivore bocaparvovirus* 3 to 5, respectively, were recently discovered in domestic cats. Firstly, the novel FBoV-1 was detected in various samples, such as feces, blood, kidney and nasal swabs, collected from asymptomatic cats in Hong Kong^1^. Thereafter, FBoV-2 and −3 were discovered during high throughput metagenomic study of fecal viromes in healthy cats^8,9^. However, neither the pathological roles of these FBoVs associated with intestinal disease nor other systemic diseases have been established. To date, the emergence of FBoVs has been reported in Belgium, China, Japan, Portugal and USA^8–13^, where the FBoV genomes were detected in the feces of cats with and without clinical signs. Later, the FBoV-1 genome was detected in cats with severe enteritis^12^, but the relationship between FBoV-1 detection and clinical presentations with its pathogenesis in infected cats is still limited. Furthermore, a recent study revealed that the FBoV-1 genome was the most co-infected virus with other viral pathogens. For example, the FBoV-1 genome was detected in the brain of feline panleukopenia virus (FPLV) infected cat showing neurological signs^10^. So far, the reports have addressed the potential role of FBoV-1 infections, yet the role of FBoV-1 when co-infected with FPLV is still unknown.

It is known that mutation accumulation and genetic recombination can both contribute to genetic diversity and virus evolution. Recombination allows viruses to quickly change their properties and results in novel genetic variants. For BoVs, genetic recombination has been focused on as a potential mechanism for virus evolution. For example, the evidence suggests that HBoV-3 emerged as a result of genetic recombination between HBoV-1 and HBoV-2^14^. Likewise, the HBoV-4 genome carried the admixture genome between HBoV-2 and HBoV-3^15–17^. Furthermore, homologous genetic recombination among CBoV-2 strains was also evident^2^. These findings indicated that genetic recombination is likely to play an important role in the diversity of BoVs.

In this study, novel FBoV-1 strains were identified in 17 FPLV-infected cats from three different households with an acute onset of depression, systemic hemorrhage, and respiratory dysfunction as well as intestinal problems. *In situ* hybridization (ISH) on three of these cats that died (one from each household) revealed a systemic FBoV-1 viral DNA with the signals localized in various cells of intestinal tissues and endothelial cells at intestinal mucosa and serosa, as well as in various lymph nodes. Genetic analysis of the full-length coding genome of the obtained Thai FBoV-1 strains indicated three separate strains (one per cat) and evidence of natural genetic recombination. The clinical presentation, through the pathological findings of FBoV-1 infected cats, is described and the potential roles of co-infection in the affected cats are addressed.

## Results

### Clinical findings

In late December 2018, all ten cats kept at household A were brought to a veterinary hospital with reported acute depression, bloody diarrhea and bloody respiratory discharge. All 10 cats, aged from 1–3 y, had been up-to-date vaccinated for FPLV, feline calicivirus (FCV), FeLV and rabies virus (RV). Later, in the beginning of January 2019, four core-vaccinated cats, aged from 1–2 y, from household B showed clinical signs of depression, followed by diarrhea, acute hemoptysis and ataxia; while three 1-month-old non-vaccinated kittens in household C were carried to the hospital in late February 2019 due to the acute onset of depression, anorexia, bloody diarrhea, hemoptysis and seizure.

Essential diagnostic tests showed severe anemia and marked leukopenia (ranging from 1,200–3,500 cells/µL) without significant changes in the blood chemistry panels in all cats. Neither protozoa nor parasitic eggs were found by microscopic fecal examination. The FeLV and FCoV antigen and FIV antibody tests all revealed negative results, while the FPLV antigen rapid test kits were positive. No evidence of detectable warfarin and organophosphate derivatives were observed in both the urine and feces samples. Bacterial cultures from nasal and fecal swabs in randomized cats from households A and B showed isolated *Klebsiella sp*. and *Escherichia coli* growth, respectively. However, no relevant findings in the aerobic bacterial cultures of the oral swab samples of cats from household C were found.

**Figure 1:**
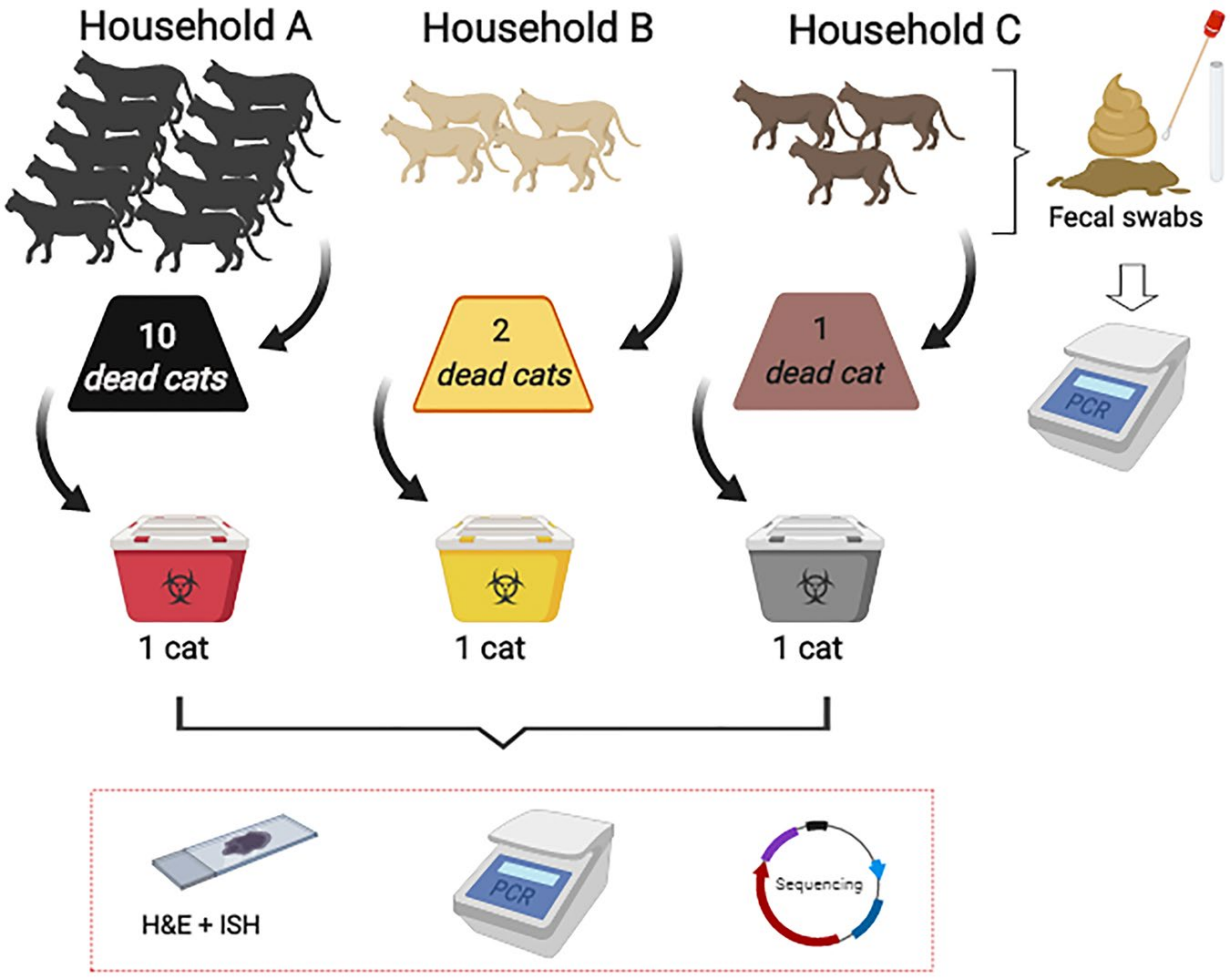
Schematic representation of FBoV-1 infected household cats and the sampling procedure. Three independent cat households (A to C) were included in this study. Fecal samples of all cats were collected for the initial virus detection using PCR panels. The number of dead cats was indicated. The dead body of one cat from each household was subjected for molecular sequencing and pathological investigations.

All 10 cats from household A did not respond to supportive treatment with antibiotics and fluid therapy, and decompensated over the course of 48 h. Including the cats and kittens in households B and C, 13 of the 17 affected cats died. Based on the availability of the owner’s consent, three of these moribund cats (one from each household, where cat no.1 to 3 was from household A to C, respectively) were submitted for necropsy and pathological examination. The FBoV-1 infected cat status, household of origin and sampling procedure are summarized in Fig. 1.

### Postmortem examination and histopathology

**Figure 2:**
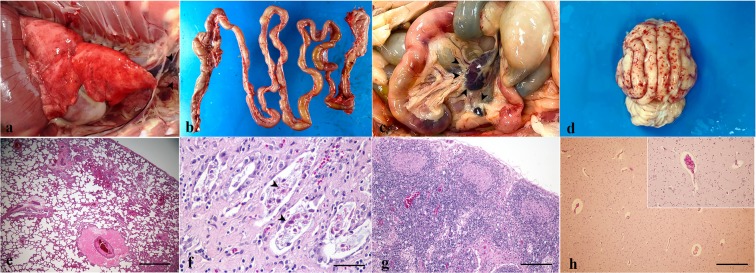
Representative photomicrographs of (**a**–**d**) gross lesions, (**e**–**h**) histopathology of the necropsied cats. (**a**) Lung, cat no. 2: severe acute diffuse pulmonary hemorrhage with severe hemorrhagic lymphadenitis of the tracheobronchial lymph node (arrowhead). (**b**,**c**) Small intestine and mesenteric lymph node, cat no. 1: severe segmental hemorrhagic enteritis with massive hemorrhagic lymphadenopathy (arrowhead). (**d**) Brain, cat no. 1: Severe acute, diffuse, petechial hemorrhagic encephalitis. (**e**) Lung, cat no. 2: severe multifocal to diffuse histio-lymphocytic interstitial pneumonia with perivascular edema (H&E stain; scale bar 850 µm). (**f**) Severe diffuse histio-lymphocytic enteritis with cryptal necrosis. Large, amphophilic, intranuclear inclusion bodies were observed in rare crypt epithelial cells (arrowhead) (H&E stain; scale bar 85 µm). (**g**) Mesenteric lymph node, cat no. 1: severe diffuse necrotic histiocytic lymphadenitis (H&E stain; scale bar 850 µm). (**h**) Brain, cat no. 1: severe diffuse expansion of the perivascular Robin-Virchow and engorged blood vessel (inset) (H&E stain; scale bar 850 µm).

The prominent gross findings of all necropsied cats revealed severe hemorrhage in various organs, including the brain, lung, liver, intestinal tract and lymph nodes. Their morphological diagnoses were severe acute diffuse pulmonary hemorrhage (Fig. 2a), segmental hemorrhagic enteritis with massive hemorrhagic lymphadenopathy (Fig. 2b,c), petechial hemorrhagic encephalitis (Fig. 2d), and necrotizing hemorrhagic hepatitis. The degree of severity of systemic hemorrhage varied among the cats. With respect to the histopathology, representative sections of the brain, lung, heart, tongue, stomach, small and large intestine, liver, spleen, and lymph nodes from three cats (no.1–3), designated 18R217C, 19R81C and 19R124C, respectively, were examined.

The morphological findings were broadly similar among all three examined moribund cats. Changes in the small intestine were severe and included extensive villous blunting and fusion along with crypt necrosis. Large, amphophilic, intranuclear inclusion bodies were observed in rare crypt epithelial cells (Fig. 2f). In the ileum, spleen, and lymph nodes, the numbers of lymphoid follicles were markedly depleted with variable accumulations of fibrin, karyorrhectic debris, and aggregates of histiocytes within the center of the remaining lymphoid follicles (Fig. 2h). In cat nos. 2 (19R81C) and 3 (19R124C), the lungs were markedly congested with multifocal to diffuse histio-lymphocytic interstitial pneumonia, and the perivascular spaces of many pulmonary vessels were markedly edematous (Fig. 2e). In cat no. 1 (18R217C), the lung was edematous, and had patchy hemorrhages with increased numbers of circulating neutrophils throughout the alveolar capillaries. In cat no. 3, the liver had random foci of hepatic necrosis with multifocal areas of lobular collapse. In addition, biliary hyperplasia was noted. In cat nos. 1 and 2, the microscopic findings of the liver were within normal histologic limits. For the heart, there were small hemorrhagic foci observed in cat no. 1.

Interestingly, cat no. 1 had vascular changes in the cerebrum and cerebellum that were characterized by engorged blood vessels containing variable numbers of PAS- and Alcian blue-negative, eosinophilic, homogenous globular material and an expansion of the perivascular Robin-Virchow space (Fig. 2i) with open spaces containing variable amounts of eosinophilic flocculent material intermixed with rare, small, proteinaceous droplets (Fig. 2i, inset). The affected blood vessels were lined by plump reactive or occasionally pyknotic endothelial cells.

### General virology

Initially, the pan-PCR panels specific for respiratory and intestinal viruses, including herpesvirus, paramyxovirus, calicivirus, coronavirus and parvovirus, were performed on the fecal extracted total viral RNA/DNA samples (n = 17). These showed positive results only with the pan-parvovirus PCR. However, the pan-BoV PCR revealed positive results in only seven fecal samples (41.2%), with three, two and two being from household A, B, and C, respectively. To complete the investigation, viral detection in fresh frozen tissue samples (rather than FFPE samples) of all three dead cats was examined by pan-viral PCRs. Positive pan-BoV PCR results were observed in the lung, lymph nodes and intestine of all cats. On the other hand, positive pan-parvovirus PCR results were noted only in the intestinal and mesenteric lymph node samples with no evidence in the other organs. Pan-PCRs for the other viruses were negative in all tissue samples.

### Detection of the FBoV-1 genome in fresh tissues of moribund cats and fecal samples of FPLV, non-FPLV and healthy cats

To overcome the sensitivity of FBoV-1 detection using the pan-BoV, specific primers targeting the FBoV-1 NS gene were used to investigate the presence of FBoV-1 genome in several tissues of necropsied cats and fecal swab samples derived from 17 FPLV-household cats, 15 non-FPLV diarrhea cats and 20 healthy cats. Specific FBoV-1 PCR revealed positive results from various organs, including the submandibular, mediastinal and mesenteric lymph nodes, lung and intestine, of all necropsied cats, as well as the brain of cat no. 1 and 3 and the liver of cat no. 3. Other fresh tissue samples were found to be negative in the FBoV-1-specific PCR tests. All 17 fecal swab samples derived from the household cats were positive for FBoV-1 by the PCR tests. The study of the presence of FBoV-1 in non-FPLV cats and healthy cats showed negative in both pan-BoV and specific FBoV-1 PCRs.

### FBoV-1 genome and phylogenetic analysis

**Figure 3:**
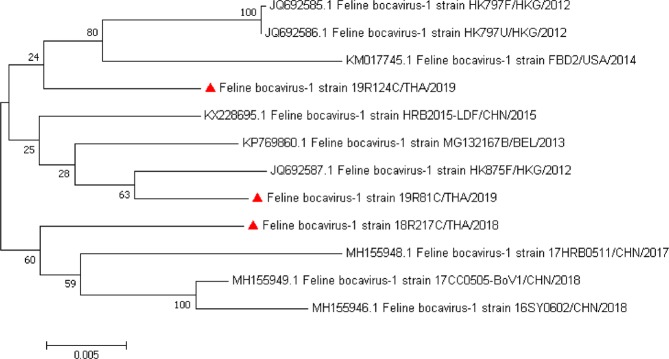
A ML phylogenetic tree showing the genetic relationship of the full-length coding FBoV-1 genomes. The ML tree was constructed using the TN93 + G model and 1,000 bootstrapping using the MEGA7 software. Bootstrap values (%) are indicated at the respective node and branch lengths are drawn to scale (number of nucleotide substitutions per site). Reference sequences used in this analysis are indicated with their respective GenBank accession no. The Thai FBoV-1 strains detected in this study are indicated by a red triangle.

**Figure 4:**
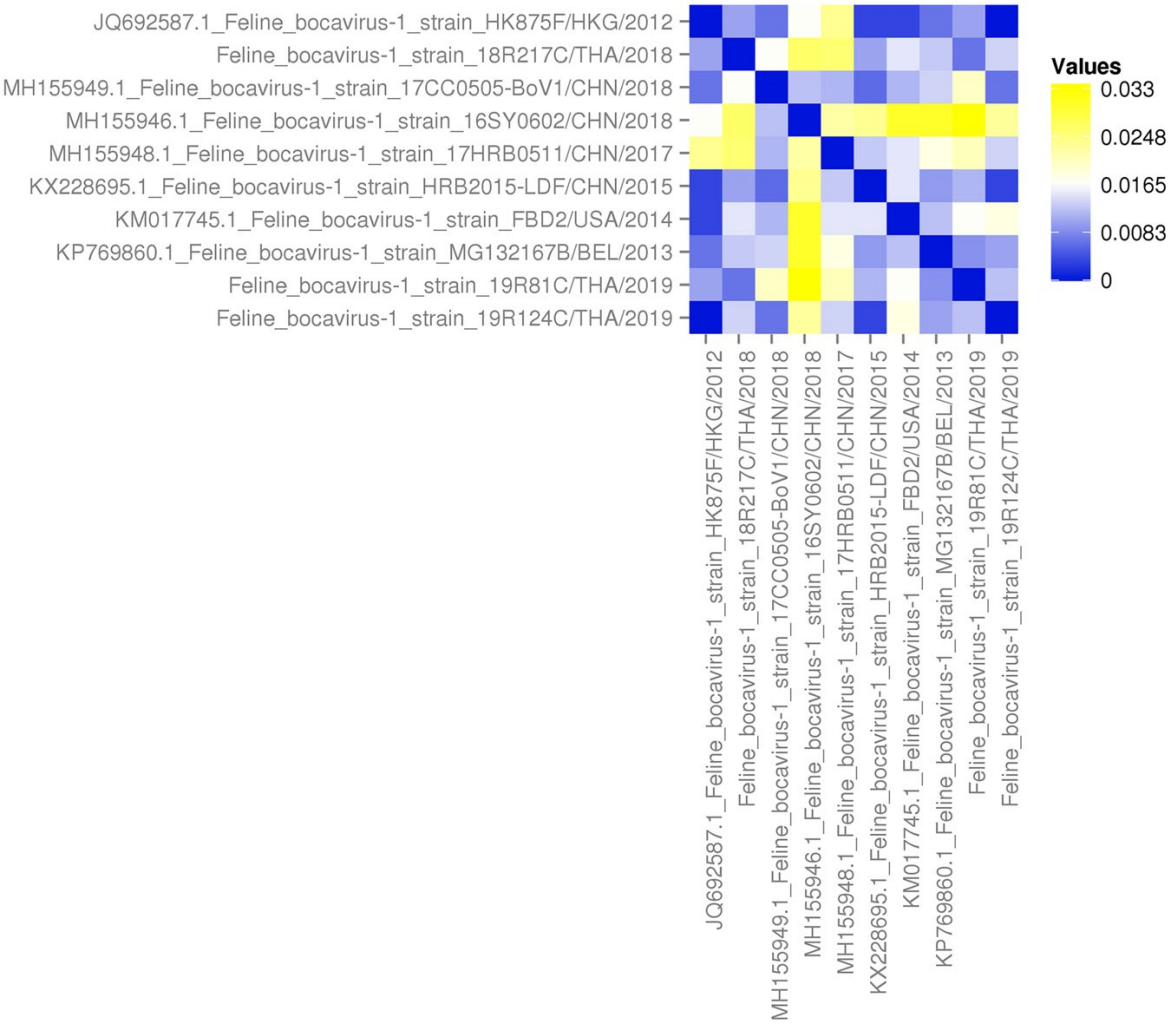
Nucleotide heatmap showing pairwise nucleotide distances among the indicated FBoV-1 strains. Values are indicated by color shading. Reference FBoV-1 sequences used in this study are indicated with their respective GenBank accession no. Schematic was generated using the Heatmapper web server (http://www.heatmapper.ca/).

**Figure 5:**
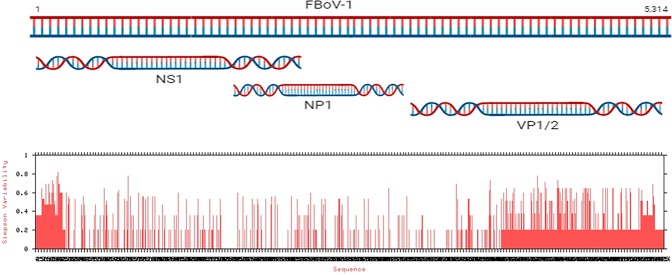
Variability of the deduced (in silico translated) full-length amino acid sequences of FBoV-1. The amino acid variability of the NS1, NP1 and VP1/2 genes among the FBoV-1 strains revealed that the VP1/2 gene is the most variable region among FBoV-1.

Whole coding genome characterization of the FBoV-1 strains detected in the lymph nodes of the three necropsied cats was performed by PCR using a panel of primer pairs to span the genome. The 5,106, 5,153 and 5,182 complete coding genomes of three novel Thai FBoV-1 strains were detecting (one strain per cat), namely strain 18R217C/THA/2018, 19R81C/THA/2019 and 19R124C/THA/2019 were recovered from cat no. 1–3, respectively, (GenBank accession nos. MN127776- MN127778). The Thai FBoV-1 strains contained three main ORFs, which encoded for the NS1 (nt 256–2670; 804 aa), the NP1 (nt 2423–3079; 218 aa), and VP1/2 (nt 3063–5201; 713 aa) proteins. Phylogenetic analysis revealed that these three Thai FBoV-1 strains were diverse and grouped into different subclades. The phylogenetic tree of FBoV-1 revealed different two main clades (1 and 2), with the 19R81C- and 19R124C/THA/2019 strains in the first clade but separated into two separate subclades (1 A and 1B), while isolate 18R217C/THA/2018 was placed in the second clade and closely related to the FBoV-1 genomes detected in China (Fig. 3). The genetic relationship among the FBoV-1 was analyzed as a pairwise nucleotide identity map (Fig. 4). The predicted amino acid sequences showed that both the NS1 and the VP1/2 regions had a high variability rate with the majority of diversity being found in the VP1/2 region (Fig. 5).

**Figure 6:**
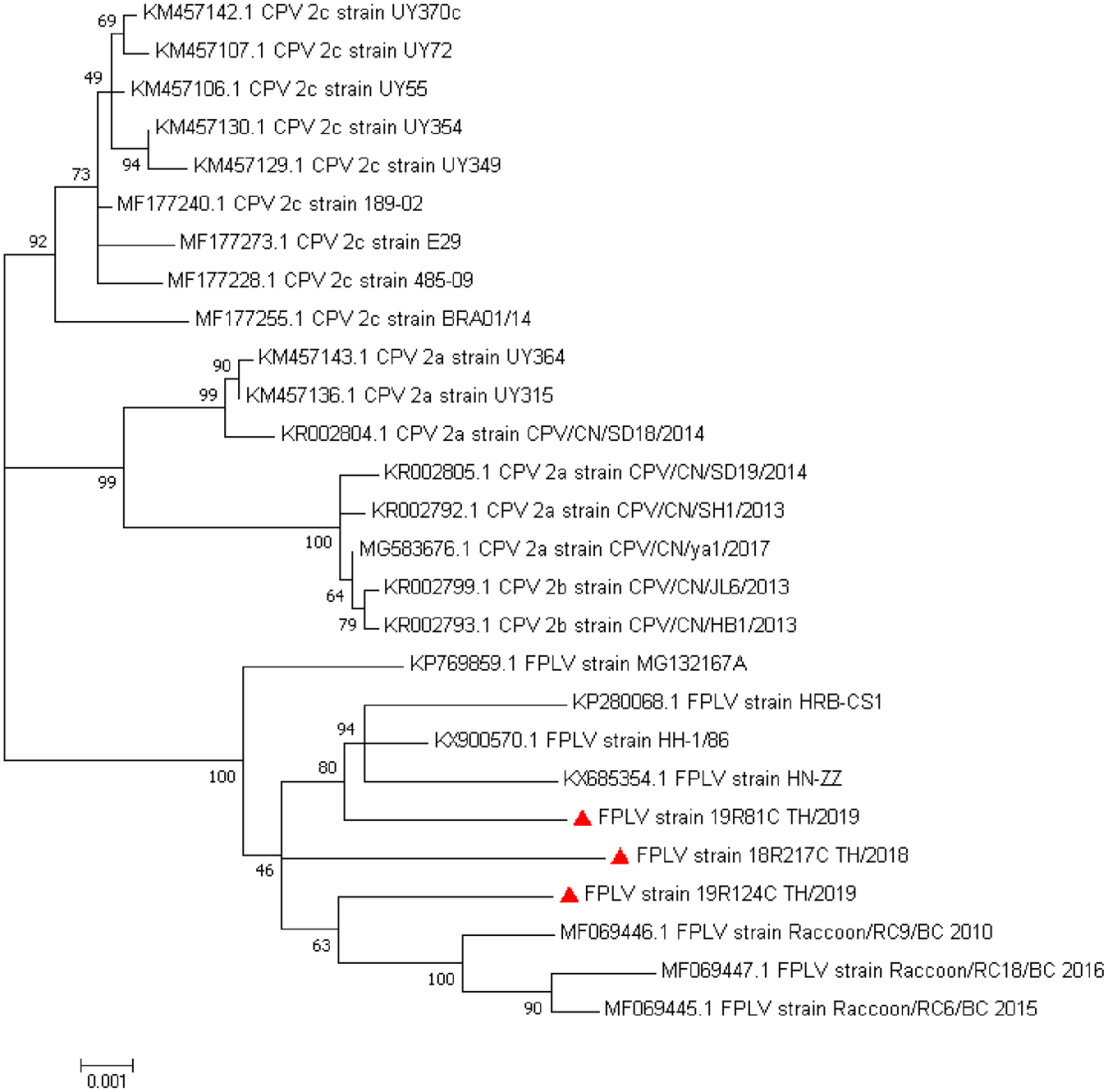
Genetic relationship, as a ML-based phylogenetic analysis of the full-length coding genome of various FPLV and CPV-2 strains. The Thai FPLV strains isolated in this study are indicated by a red triangle.

The PCR analysis for FPLV revealed the presence of the FPLV genomes in the intestinal samples of cat nos. 1–3. Subsequent PCR amplification with designed primers to span the FPLV genome allowed the full-length coding genome sequences of the Thai FPLV strains from the three cats (nos. 1–3), to be obtained, and were designated FPLV 18R217C/THA/2018, 19R81C/THA/2019 and 19R124C/THA/2019 for that from cat no. 1 to 3, respectively, (GenBank accession no. MN127779- MN127781). The ML phylogenetic analysis of the complete coding genomes of these Thai FPLV strains is shown in Fig. 6.

### Genetic recombination in one of the Thai FBoV-1 strains

With respect to the genetic recombination forced evolution of FBoV-1, two genetic recombination detections modules annotated the FBoV-1 18R217C/THA/2018 isolate as a potential recombinant virus. The RDP software package revealed evidence of genetic recombination in the NS1 region of this strain and this was supported by a number of statistical measurement assays, including the RDP, GENECONV, BootScan, Maxchi, Chimaera, SiScan and 3Seq with a *p*-value of 1.838 × 10^−6^, 3.435 × 10^−3^, 1.641 × 10^−6^, 1.795 × 10^−10^, 1.371 × 10^−9^, 1.077 × 10^−2^, and 1.041 × 10^−11^, respectively. Among the FBoV-1 genomes available in GenBank, FBoV-1 strain HK875F (JQ692587) isolated in Hong Kong and strain 16SY0602 (MH155946) from China served as the putative major and minor parents, respectively.

**Figure 7:**
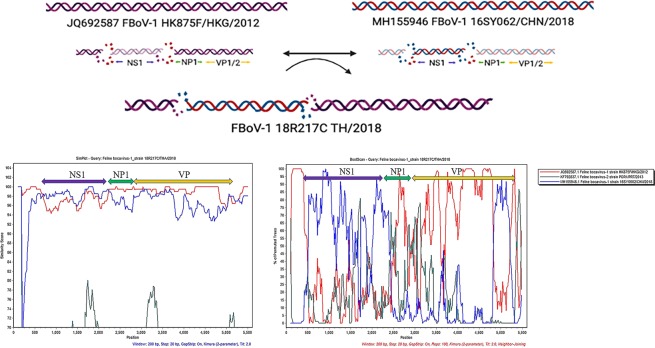
Schematic diagram of the naturally recombinant FBoV-1 18R217C/THA/2018 strain. FBoV-1 HK875F (JQ692587) isolated in Hong Kong and FBoV-1 16SY062 (MH155946) from China served as the putative major and minor parents. The potential recombination event was detected in the NS1 gene and was supported by similarity and bootscan analysis, which indicated that FBoV-1 HK875F (red line) served as a template at the beginning of the NS1, then the genome sequence was replaced by the sequence from the FBoV-1 16SY062 until the beginning of the NP gene. The FBoV-1 18R217C/THA/2018 isolate served as the query. The y-axis indicated the percentage of nucleotide identity and permutated trees for similarity plot and boot scanning, respectively, within a 200 bp-wide window with a 20-bp step size between plots.

The obtained RDP results were then further analyzed to identify the recombination breakpoints using the SimPlot software, which also supported that FBoV-1 18R217C/THA/2018 was recombinant and that the recombination breakpoint occurred in the NS1 coding region (Fig. 7). The generated similarity plot for the FBoV-1 18R217C/THA/2018 isolate showed a high nucleotide identity to the 16SY0602 isolate (blue line) at the beginning through to the end of the NS1 region. Similarly, the bootscan analysis confirmed the preliminary results of the RDPs and similarity plot by unveiling the evidence of the sequences derived from the FBoV-1 strain 16SY0602 inserted at nt 714 to nt 2,268 within the NS1 gene of the 18R217C/THA/2018 strain. The FBoV-1 strain HK875F served as a parental template at the beginning of NS1 gene and the whole NP1 through the VP1/2 genes (red line). No evidence of a potential recombination breakpoint was observed in the other two Thai FBoV-1 strains nor in the three Thai FPLV strains of this study.

### Viral detection and tissue tropism determined by ISH and IHC analyses

#### FBoV-1 localization in tissues

**Figure 8:**
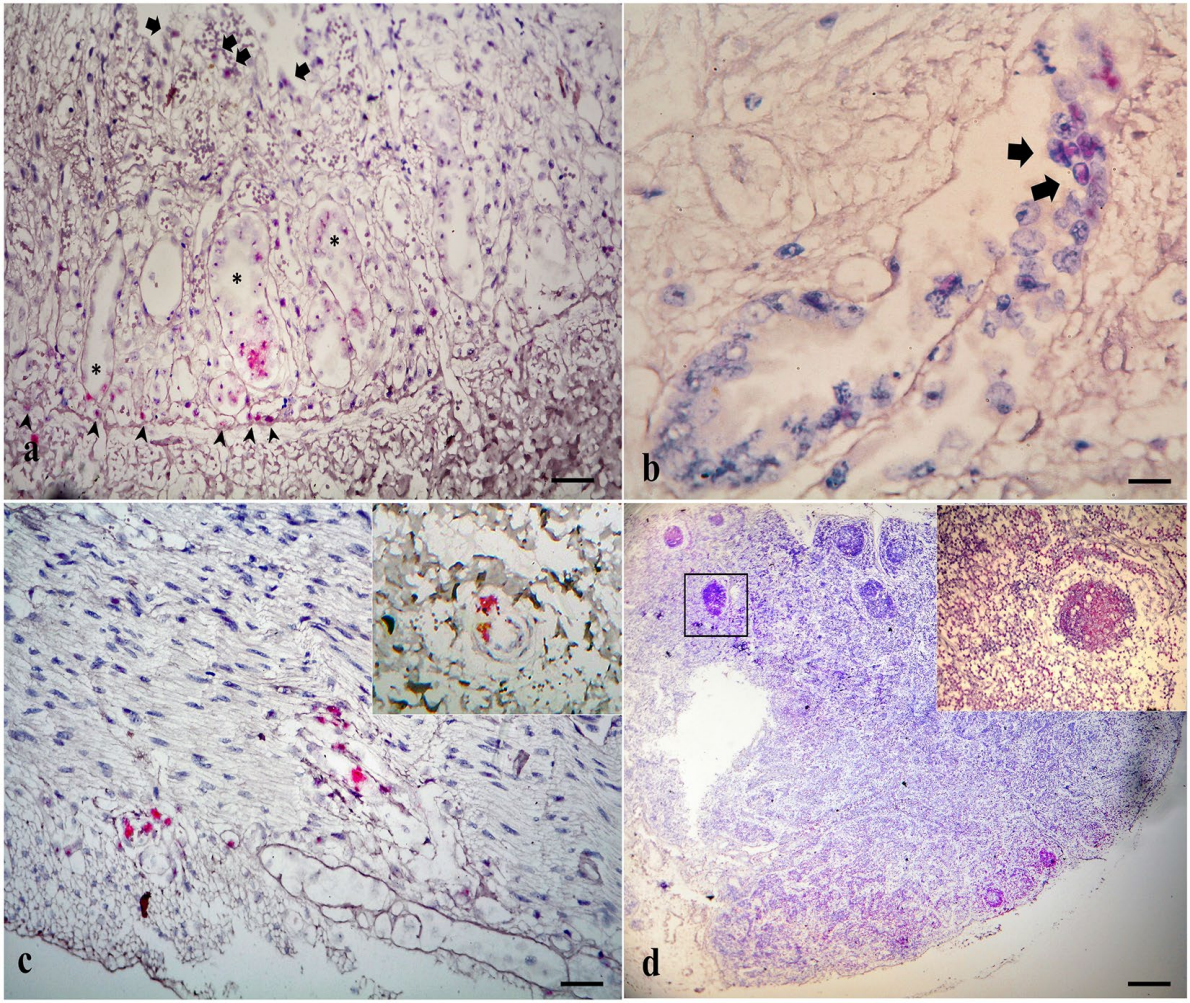
Representative photomicrographs of tissues from cat nos. 1–3 with ISH detection of FBoV-1 antigens. FBoV-1 signals were evident in the intestine and lymph nodes. (**a**,**b**) Small intestine, cat no. 3: FBoV-1 DNA signals were strongly localized in the nucleus of cryptal epithelial cells (asterisk) and stromal supporting cells (arrowheads) with rare localization in epithelial cells lining the villi (arrows); scale bar, 170 µm. (**c**) The FBoV-1 signals localized in endothelium of small-sized blood vessels at intestinal serosa and the junction between submucosa and lamina propria (inset); scale bar, 170 µm. (**d**) Submandibular lymph node, cat no 1: FBoV-1 nucleic acid signals were diffusely localized in lymphoid follicles, and most of the cells were lymphocytes and histiocytes (inset); scale bar, 850 µm.

To investigate the topographic distribution and tissue tropism of FBoV-1, the FBoV-1 specific ISH was performed in FFPE tissues derived from all the necropsied cats. Strong viral nucleic signals were strongly labelled in nucleus of various intestinal and lymphoid cells, including cryptal epithelial cells, intestinal stromal supporting cells, infiltrating lymphocytes and weak labelled in enterocytes (Fig. 8a,b). Furthermore, FBoV-1 nucleic acid signals were localized in the endothelial cells of small blood vessels at the conjunction between submucosa and lamina propria, and at the intestinal serosa (Fig. 8c). Moreover, the FBoV-1 nucleic acid signals were detected nucleus of lymphoid cells locating in submandibular, mediastinal and mesenteric lymph nodes. Consistent with these histopathologic findings, all sections of the submandibular, mediastinal and mesenteric lymph nodes of all cats revealed a prominent FBoV-1 ISH signal in the nucleus and cytoplasm of lymphocytes and histocytes, within the cortex and subcapsular sinus of lymph nodes (Fig. 8d). The FBoV-1 antigenic signals were not evident in other examined tissues. The negative controls were all negative (Supplementary Fig. 1).

#### FPLV localization in tissues

**Figure 9:**
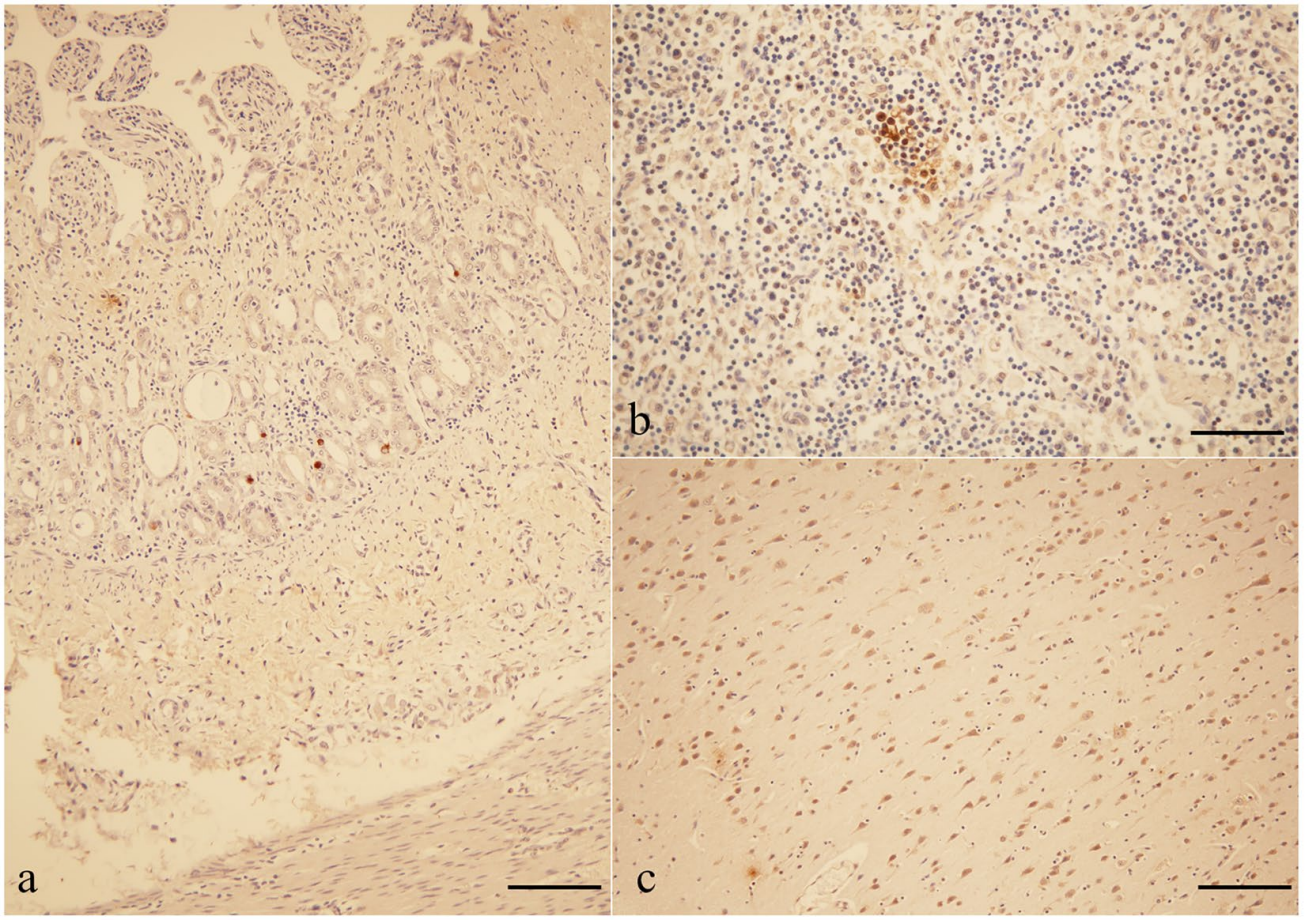
Representative photomicrographs of tissues from cat nos. 1–3 with IHC detection of FPLV antigens. (**a**) Intestine, cat no. 3: FPLV was detected in rare cryptal epithelial cells. Scale bar, 170 µm. (**b**) Mesenteric lymph node, cat no. 1: FPLV was detected in scattered lymphoid cells with strongly positive immunoreactivity for FPLV in the cytoplasm of the cells. Scale bar, 85 µm. (**c**) Cerebrum, cat no. 2: FPLV were frequently observed in the cytoplasm of neurons. Scale bar, 170 µm.

To illustrate the FPLV localization and associated lesions potentially caused by the virus, IHC was performed in the FFPE tissues of all necropsied cats. The IHC staining confirmed that the FPLV viral antigens were detected in the rare cryptal epithelial cells that contained an eosinophilic intranuclear inclusion and in lymphocytes through the GAL tissues (Fig. 9a). Scattered lymphoid cells in the mesenteric lymph node showed a strong positive immunoreactivity for FPLV (Fig. 9b), while the other lymph nodes were immuno-negative. In cat no. 2 and 3, the FPLV specific proteins were also detected by IHC analysis in most of the neuronal cells of both the cerebrum and cerebellum, while cat no. 1 revealed weaker staining in the cerebrum and cerebellum than the other two cats (Fig. 9c). The FPLV-specific IHC staining was not evident in other tissues.

## Discussion

Outbreaks of hemorrhagic enteritis were presented in three different household cat colonies under well-managed and environmental-closed systems. The disease spread to neighboring cats causing fatality in 13/17 symptomatic cats within a day. Initially, FPLV was thought to be the primary infectious agent in all cases. However, clinical presentations of hemorrhagic syndrome with multi-systemic involvement are atypical findings for simple FPLV infections. Thus, an unknown concurrent infectious etiology, highly likely viral, may have played an important role in these outbreaks. This prompted us to expand the search for other unknown pathogens. Interestingly, we identified a FBoV-1 co-infection in these cats, arguing the potential role of the both viruses in the outbreaks.

Little is known about the clinical significance of FBoV-1, and so it is discussed based on the data from previous studies in other bocaparvoviruses. The FBoV-1 viral genomes have been previously detected in both cats with and without diarrhea^1,11,12^, but the pathological roles and viral localization are still debatable. In this study, the presence of FBoV-1 associated with these outbreaks was confirmed by the PCR from various fresh tissues of the moribund cats and fecal swabs from cats in the same households. Furthermore, the FBoV-1 localization and distribution were documented by ISH analysis, suggesting the extent of infection and viral tropism. Detection of FBoV-1 DNA using PCR showed the patterns of viral distribution. However, the demonstration of FBoV-1 in and other organs of moribund cats by PCR, in the absence of FBoV-1 ISH signal outside the intestinal tract and lymphoid tissues, was inconsistent, suggesting the possibility of viral spread via the blood stream. This finding was consistent with previous publications that documented the evidence of bocaviral viremia in the absence of viral localizations^2,18^. Since various BoVs were identified in intestinal tract of humans and other animals, with and without clinical diseases, investigations of FBoV-1 were recently conducted in intestinal tract samples of cats^1,8,9,11–13^. However, the role of FBoV-1 enteric disease is not fully yet investigated. To better understanding the role of FBoV-1 associated with intestinal lesions, substantiation of virus localization in tissue and association of histological features are important. In the present of study, the FBoV-1 genomic signals were localized mainly in the nucleus of cryptal epithelium, intestinal stromal supporting cells and rarely detected in epithelium of villi. This finding indicated the first identification of FBoV-1 localization in the intestinal tissues and supported the previous evidences suggesting the FBoV-1 is more prevalent in fecal samples. This finding also indicated the common features of bocaparvoviral infection associated with intestinal diseases. Furthermore, the FBoV-1 signals were also detected in endothelium of small-sized blood vessels at intestinal submucosa and serosa, suggesting another possible evidence of FBoV-1 spread via the blood stream. Of note, the detection of FBoV-1 in many tissues would argue it is possible to be a persistent virus in cats, whether in blood or specifically in the tissues, as recent studies of persistent infection of BoVs^19–21^. Therefore, in this present study, a contributory role of FBoV-1 enteric disease is possible while a role of FBoV-1 viremia is needed further studies of FBoV-1 pathogenesis.

The FPLV antigens were consistently detected by IHC within the necrotic crypts in the intestine as well as the FBoV-1 signals detecting by ISH. The presence of the FBoV-1 and FPLV antigens in the necrotic crypts along with the histologic features of amphophilic intranuclear inclusion bodies in the crypt epithelial cells, crypt loss, and collapse of mucosal architecture supports the clinical presentation of diarrhea and gross findings consistent with a diagnosis of viral enteritis. However, the FPLV antigen was only detected at a low level in the mesenteric lymphoid tissue, while FBoV-1 antigens were detected at a large-scale, particularly in the necrotic areas of both the cortex and medulla of the lymph nodes. The presence of FBoV-1 antigens was not limited to mesenteric lymph nodes but was also systemically spread through the various lymphoid organs. The presence of FPLV and FBoV-1 antigens may also reflect the time point of infection, as the FPLV antigen might be cleared after necrosis of the lymphoid tissue. In addition, FBoV-1 may be associated with the lymphoid necrosis itself similar to reports of bocapavovirus infection in various host species^18,22^. These results support that the pathological role of FBoV-1 can be associated with lymphoid necrosis, as reported previously, and may contribute to the acquired immunodeficient state of the host^18,23,24^. The role of immunocompromise situation, as FPLV infection in theses cats, may induce secondary viremia or *vice versa*. As such, hosts with FBoV infection are likely to be more susceptible to other infectious diseases. Subsequent systemic immunosuppression might allow the virulent bacteria to colonize in tissues and, therefore, contributed to lesions in other organs. *In vivo* study of the FBoV-1 infection may demonstrate the exact role of FBoV-1 in the focused diseases.

In this study, the possibility of a synergistic relationship between FPLV and FBoV-1 was hypothesized. A recent study showed that BoV can induce a DNA damage response (DDR) for cellular infection^20,25–27^, and the fact that parvovirus has limited its genetic resource and requires the DDR process to facilitate its replication process^26,28^. Thus, the association of BoV infection with enhanced parvovirus replication has been reported^29^, while vice versa, parvovirus can also induce the DDR process^26,28^ and help BoV infection^27^. Thus, we hypothesized the possible synergistic effect of FPLV and FBoV-1 infection in this study. Alternatively, many current studies have revealed that BoV causes not only acute infections but also probable persistent or subclinical infections by forming the covalently closed circular DNA (cccDNA) structure and integrating into the host genome^21,30,31^. Furthermore, the BoVs have been associated with lymphoid necrosis^18,23,24^ and may be a primary persistent infection resulting in immunosuppression, allowing secondary infection with FPLV, despite previous vaccination. In this study, the role of FBoV-1 infections remains elusive, whether primary or secondary. Nevertheless, experimental study of such dual infections is needed to further clarify the true significance of the FBoV-1 infections.

Upon a retrospective FBoV-1 investigation on fecal samples of healthy and non-FPLV diarrhea cats with no FBoV-1 genome could be detected, suggesting that FBoV-1 is not usually a primary cause of enteritis. Since a temporal study with only a relatively small number of cats was conducted, no definitive conclusions could be made.

Molecular genetic analysis suggested that these three Thai FBoVs-1 strains are diverse and disperse, as shown by grouping in three different clusters (clades) in the phylogenetic topology. Preliminary analysis of the full-length coding genome of FBoV-1 strains, including these three Thai FBoV-1 strains, revealed an interesting phylogenetic topology with two main clades and all three Thai strains placed in different subclades (clades 1 A, 1B and 2). Thereafter, we analyzed the phylogeny based on individual nonstructural and structural genes, which revealed discordant phylogenetic patterns between the different genes, suggesting potential homologous recombination events contributing to the increase genetic diversity.

Here, we identified the genetic recombination breakpoint as occurring in the NS1 coding region of the FBoV-1 18R217C/THA/2018 strain. Even though the NS1 gene is more conserved among the BoVs^32^, the genetic recombination breakpoints identified in this region have been reported before^33,34^. Because the NS1 gene plays a role in viral replication, modulation of the innate host immune response^4,35,36^, and association with cell cycle arrest^37^, our findings not only indicated and supported that genetic recombination plays an important role in the diversity of BoVs^2,14–17^ but also hypothesized the mutation of NS1 might be associated with FBoV-1 pathogenesis.

In summary, we present the first comprehensive clinical and pathological description through genomic analysis of three circulating Thai FBoV-1 strains that were detected during an outbreak of hemorrhagic disease in three different households of cats. Although the role of FBoV-1 associated with systemic hemorrhage of these cats remained undetermined, a contributory role of FBoV-1 associated with enteric disease is possible. Since antigens/nucleic acid of the FPLV, but not those of other viruses, were detected in tissues of diseased cats, the clinical presentations are likely synergistic effects of dual infection with FPLV and FBoV-1, arguing the causative role of FBoV1 in the disease progression. Thus, we speculate the synergistic effects of dual infections with FBoV-1 and FPLV, ultimately resulting in a more severe and sudden clinical disease. The genetic recombination of FBoV-1 is firstly identified in this study, supporting the evolutionary mechanism of BoVs. Further in-depth studies of the pathology of the FBoV-1 infection are required to fully elucidate the significant clinical presentation of infected cats and to provide optimal guidance for diagnosis, surveillance and quarantine of FBoV-1 suspected cats. Further applied studies should be undertaken into FBoV-1 protein functions, to better understand how recombination events alter FBoV-1 pathogenesis and transmission.

## Materials and Methods

### Animals and sample preparations

A total of 17 domestic cats (*Felis catus*) from three different, independent households (10, four and three cats from household A, B, and C, respectively) locating in Bangkok metropolitan, Thailand, showed a similar acute onset of depression followed by bloody diarrhea, hemoptysis and seizure. The beginning of the clinical presentation was evident within 24 h after recognition of the animal’s depression. Initial diagnostic tests were performed on all cats, including hematology, serum chemistry profiles and fecal examination, upon their hospital visit. The antigen of FPLV, feline leukemia virus (FeLV) and feline coronavirus (FCoV), and antibody of feline immunodeficiency virus (FIV) were screened for using commercial test kits (Rapid Anigen, Bionote, South Korea). General toxicology, including warfarin and organophosphate poisoning, detection from urine and feces, and bacterial cultures from nasal and/or fecal swabs were performed in randomized cats. During supportive treatment, fecal swabs were further collected in all cats for further investigation. All experimental protocols were approved by the Chulalongkorn University Animal Care and Use Committee (No. 1631002). All procedures were done in accordance with the relevant guidelines and regulations. The dog owners gave his/her written consent for sample collection and data publication.

Later, a total of 13 of these cats died (10, two and one from household A, B, and C, respectively). With permission of the owners, three of the 13 moribund cats (one from each household) were submitted for necropsy at Department of Pathology, Faculty of Veterinary Science, Chulalongkorn University. Selected tissue samples, including the brain, lung, heart, stomach, small and large intestine, liver, spleen, lymph nodes, and kidney, were collected, fixed in 10% neutral buffered formalin, and submitted for routine histopathology plus Periodic acid-Schiff (PAS) and Alcian blue staining. Microscopic findings were evaluated by an American board-certified veterinary pathologist (TK). Moreover, fresh tissues, including the brain, trachea, lung, thymus, submandibular and mesenteric lymph nodes, liver, and intestines, were harvested for further molecular analysis. Fecal swabs from all 17 cats and fresh tissues from the three necropsied cats were separately homogenized and total viral RNA/DNA was extracted using a viral nucleic acid extraction II kit (Geneaid, Taipei, Taiwan). The quality and quantity of extracted RNA/DNA was determined by absorbance of A260/280 ratio using a spectrophotometer (NanoDrop, Thermo Scientific™, USA). Pan-respiratory and intestinal virologic PCR panels, including detection of herpesvirus^38^, paramyxovirus^39^, parvovirus^40^, coronavirus^41^, and BoVs^1^, were performed in selected fresh tissue samples (lung, lymph nodes, and intestine) derived from three necropsied cats. The pan-calicivirus-specific PCR primers; forward (CV2429F): 5′GAACTACCCGCCAAT3′ and reverse (CV2550R): 5′AGCACRYCATATGCGGC3′, were designed from alignment of the ORF1 sequences of various caliciviruses and then performed in the same samples.

To establish the prevalence of the FBoV-1 in healthy cats and non-FPLV cats showing diarrhea, fecal swab samples from twenty healthy and fifteen fecal samples from non-FPLV cats locating in the same area (Bangkok, Thailand) of the outbreaks were included for FBoV-1 detection.

### Genomic identification and characterization of the FBoV-1 and FPLV strains

For initial identification of FBoV, the positive pan-BoVs PCR samples were then sequenced in order to confirm and classify the BoV origin. The sequencing analysis confirmed the validity of the positive pan-BoV PCR samples and so the presence of FBoV-1. To increase the sensitivity of FBoV-1 detection and to investigate the presence of the FBoV-1 genome in other organs of the three moribund cats and to establish the outbreak of disease in neighboring cats, the extracted total viral RNA/DNA derived from selective fresh tissue samples of the three necropsied cats, the fecal swabs from all FPLV-cats, non-FPLV cats and healthy cats were tested by FBoV-1-NS1-specific PCR using the primers as described previously^1^.

Furthermore, degenerated primer sets specific for the FBoV-1 genome were designed by alignment of various FBoV-1 strains available from the NCBI GenBank database, to allow PCR amplification and commercial sequencing to obtain the complete coding genome the Thai FBoV-1 strains (Supplementary Table S1). Briefly, the total viral RNA/DNA derived from the lymph nodes of three necropsied cats were individually amplified using a Qiagen OneStep RT-PCR kit (Qiagen GmbH, Hilden, Germany) with a combination of reverse transcriptase and DNA polymerase, and specific primers. The amplicons were generated by thermocycling at 50 °C for 30 min, 94 °C for 15 min and then 40 cycles of 94 °C for 30 s, 50 °C for 30 s and 72 °C for 1 min, before a final 72 °C for 7 min. The PCR products were visualized on 1% (w/w) agarose gel electrophoresis and further purified using a Monarch DNA Gel extraction kit (New England Biolab, Frankfurt, Germany) prior to commercial Sanger sequencing (Macrogen Inc, Seoul, South Korea). The complete coding sequences of the FBoV-1 isolates obtained from the three necropsied cats were deposited in GenBank (accession numbers MN127776-MN127778).

In addition, primers specific for the FPLV VP1/2 gene were retrieved from a previous publication^40^ and the primers for the FPLV NS1 and NP1 genes (Supplementary Table S2) were designed by alignment of the available FPLV genomes in GenBank to amplify the complete coding genome of the obtained FPLV strains in this study. Briefly, the total RNA/DNA extracted from intestinal samples of the three necropsied cats, and fecal samples derived from symptomatic and healthy cats was individually amplified using a Qiagen OneStep RT-PCR kit and amplified and sequenced as mentioned above for FBoV-1, except for the thermal cycling annealing (55 °C for 2 min) and extension (72 °C for 2 min) steps. The complete coding sequences of the Thai FPLV strains were deposited in GenBank (accession numbers MN127779-MN127781).

### Sequencing and phylogenetic analysis of FBoV-1 and FPLV

The complete coding genome sequences of the FBoV-1 Thai strains were aligned with the published FBoV-1 sequences using the MAFFT version 7 (http://mafft.cbrc.jp/alignment/server/) and MEGA7 software. The output sequence alignments were then used as a template for phylogenetic tree construction. The phylogram was constructed using the maximum likelihood (ML) method with the TN93 + G model, which was selected using the find-best-fit model algorithm in Mega7 according to Bayesian Information Criterion. The tree was tested with 1,000 bootstrapping replicates. Sequence pairwise distances between the FBoV-1 genomes were calculated using the Maximum Composite Likelihood model and their evolutionary distance was analyzed in MEGA7. The output pairwise nucleotide distances were generated and visualized in a pairwise heat map, using the Heatmapper web server (http://www.heatmapper.ca/)^42^. Variability in the predicted (in silico translated) amino acid sequences of the NS1, VP1/2 and NP1 genes of the FBoV-1 strains were analyzed by the Simpson diversity index using the online Protein Variability Server software (http://imed.med.ucm.es/PVS/).

For the FPLV genomes derived from the affected cats, phylogenetic analysis was conducted using the same criteria for the FBoV-1 genomes above. The ML phylogenetic tree of the FPLV genomes was constructed using a general reversible model and bootstrapped with 1,000 replicates.

### Genetic recombination in a Thai FBoV-1 strain and FPLV strains

Potential genetic recombination in the obtained Thai FBoV-1 strain was suspected from the phylogenetic analysis and so was examined for using the integrated recombination detection program 4 (RDP4) package v. Beta 4.94 software^43^. A range of recombination detection methods, including Bootscan, Chimera, GeneConv, MaxChi, RDP, SiScan, and 3Seq, with a cut-off acceptable *p*-value of 0.01, were systematically used for the analysis. Bonferroni correction was set as the default setting. The alignment of each detected FBoV-1 sequence with those available in GenBank was used as the template for genetic recombination analysis. Any potential recombination event that was detected by at least four methods was considered to be a potential recombinant^44,45^. Major and minor parents of the potential recombinant detected in the RDP were further analyzed using a similarity plot and bootscan analysis in the SimPlot software package v. 3.5.1^46^. The recombinant genome derived from the RDP analysis was used as the query sequence. The recombination analysis was conducted with a window size of 200 bp and step size of 20 bp. The recombination breakpoints were identified and analyzed by the Kimura-2 parameter (K2P) model for bootscan analysis and the GapStrip model for the similarity plot. A phylogeny of the detected recombination region was then constructed in order to confirm the suggested recombination. The ML phylogenetic tree construction criteria and procedure were conducted as described in the analysis of the complete coding genome. For complete analysis, the other Thai FBoV strains and all the Thai FPLV strains obtained in this study were also screened for potential recombination in a similar approach as that described above for FBoV-1.

### Tissue distribution of FBoV-1 nucleic acids

The distribution of FBoV-1, and hence virus tropism, was evaluated by ISH to confirm the presence of viral DNA in formalin-fixed paraffin-embedded (FFPE) samples from the three necropsied cats. Examined tissues included the brain, lung, heart, tongue, stomach, small and large intestine, liver, spleen, and lymph nodes. The DNA probe covering 133 bp of the NS1 gene of FBoV-1 was prepared using a PCR DIG Probe Synthesis Kit (Roche Diagnostics, Basel, Switzerland), according to the manufacturer’s instructions. The PCR reaction was performed under the same thermal cycling conditions described earlier using the described NS1 primers^1^ except using the digoxigenin (DIG)-labeled oligonucleotides instead of the unlabeled oligonucleotides. Validation of the hybridization probe was performed by size resolution on 1.5% (w/v) agarose gel electrophoresis and using control DNA for Northern blot immunolabelling (not shown). The ISH with chromogenic DNA was performed as previously reported^47^, with minor modification. Briefly, each 4-µm-thick FFPE slide was deparaffinized, rehydrated and subsequently rinsed in phosphate-buffered saline (PBS). Thereafter, the slides were treated with proteinase K (200 µg/mL; VWR, Radnor, USA) in prewarmed PBS at 37 °C for 15 min. Slides were then washed three times in PBS for 5 min each. In order to eliminate the endogenous alkaline phosphatase, cold 20% (v/v) acetic acid were applied on each slid for 20 sec, and the slides were soaked with PBD 3 times for 5 min each then dried at room temperature. Thereafter, all slides were then prehybridized in hybridization buffer [50% (v/v) formamide, 6X SSC, 5X Denhardt’s solution, 100 µg/mL DNA, and 0.5% (w/v) sodium dodecyl sulfate] at 37 °C for 45 min. Meanwhile place on ice. Fifty ul of hybridization buffer containing probes for FBoV-1 were applied on each slide while hybridization buffer containing DIG-labeled feline herpesvirus probe was placed on each negative control slide. The slides were then transferred to a humidified chamber and further incubated at 37 °C for overnight. Then slides were soaked in the series of buffer standard saline citrate including 2X SSC at pH 7.0–7.4 and 1X SSC at 37 °C for 5 min of each and 0.5X SSC at 37 °C for 30 min. For blocking nonspecific binding, all slides were flooded with blocking solution mixture composed of 1 ml blocking solution and 9 ml of Maleic acid buffer (DIG Wash and Block Buffer Set, Roche, Basel, Switzerland), then were incubated at room temperature for 60 min. The slides were subsequently rinsed by 20 mM Tris-buffered saline (TBS) at 37 °C for 5 min. After nonspecific blocking, 100 ul of anti-DIG-AP Fab fragments (Roche, Basel, Switzerland) (1:200 in 1X Blocking solution) were placed on each slides, and the slides were incubated in the moist chamber for 60 min at room temperature, then washed three times with TBS for 5 min each, and placed in detection buffer (DIG Wash and Block Buffer Set, Roche, Basel, Switzerland) for 5 min. Liquid permanent red (LPR) (Dako, Glostrup, Denmark) was applied in the dark room at room temperature for 30 min. Slides were then counterstained with hematoxylin, dehydrated, and coverslips applied.

The brain, lung, liver, lymph node and intestine sections from cat no. 1 with an unrelated probe specific glycoprotein B of feline herpesvirus incubation were used as negative controls and the tissues of brain, lung and lymph node derived from a FBoV-1 negative cat served as additional negative controls.

### Staining for FPLV by immunohistochemistry (IHC)

All FFPE tissues from the three necropsied cats were subjected to IHC analysis using the 1.B.450 mouse monoclonal anti-canine parvovirus antibody (Abcam AB59832, Cambridge, UK) as the primary antibody. Briefly, after deparaffinization and rehydration, tissue sections were pretreated by 0.1% (w/v) trypsin at 37 °C for 25 min, followed by blocking endogenous peroxidase activity with 5% (w/v) skim milk at 37 °C for 40 min. After washing three times with PBS, the sections were incubated with primary antibody (1:200 dilution) at 4 °C overnight followed by detection using the Dako REAL EnVision Detection System (Dako, Glostrup, Denmark) at RT for 45 min. After triplicate washings with PBS, a positive antigen-antibody reaction was observed by labeling with DAB and counterstained with Mayer’s hematoxylin. The FPLV-positive intestinal tissue from a PCR-positive FPLV-infected cat served as a positive control. Samples treated with distilled water (DW) instead of the primary antibody and non FPLV-infected samples served as negative controls.

### Ethics Statement

All experimental protocols were approved by the Chulalongkorn University Animal Care and Use Committee (No. 1631002). All procedures were done in accordance with the relevant guidelines and regulations. The cat owners gave his/her written consent for sample collection and data publication.

## Supplementary information

Supplementary information

## Data Availability

All the data supporting our findings is contained within the manuscript. Three full-length coding FBoV-1 and FPLV sequences have been deposited in NCBI GenBank under accession numbers MN127776 – MN127781.
