# Supplementary material for: Feline bocavirus-1 associated with outbreaks of hemorrhagic enteritis in household cats: potential first evidence of a pathological role, viral tropism and natural genetic recombination
Source: Sci Rep. 2019 Nov 8;9:16367. doi: 10.1038/s41598-019-52902-2 (PMC6841677; doi:10.1038/s41598-019-52902-2)
Supplement: Supplementary file 1 — Supplementary information [file 41598_2019_52902_MOESM1_ESM.pdf]

Feline bocavirus-1 associated with outbreaks of hemorrhagic enteritis in household cats: potential first evidence of a pathological role, viral tropism and natural genetic recombination

Chutchai Piewbang,[1] Tanit Kasantikul,[2] Kidsadagon Pringproa,[3] Somporn Techangamsuwan,[1,4,*]

Supplementary materials

Supplementary Table S1 The FBoV-1-specific primers used for the full-length genome sequencing. The primers were designed based on alignment of multiple FBoV-1 genomes available in GenBank.

Primer name	Sequence (5'-3')
**FBoV1_55F**	CYGGCGCGATGACGTGTCAG
**FBoV1_577R**	GCGCCGCATAGCAGACYTCAG
**FBoV1-NS1F***	TTTGGGGCTGARGTCTGCTATGC
**FBoV1-NS1R**	ATGCGGCTGAGRTGTACCTTGACC
**FBoV1-1139F**	TCCTAGTCAGGGACGAACCAATC
**FBoV1-1596R**	GACACAGCAGATCCAATGTCCCA
**FBoV1-2245R***	CGATGTCGCCTCGATCGTGYAGTTC
**FBoV1-2177F**	ATGAGACCGGAACGTGTATGAGCT
**FBoV1-3583R**	GGCGCCCCCTCTTGTCCAT
**FBoV1-3553F**	AGACGGCTGGGGATGGACAAGA
**FBoV1-4963R**	GGYAGTGTGCCATCAGCCGAATC
**FBoV1-4734F**	ATGGCTAGATGGGGTTCAGTG
**FBoV1-5368R**	GTAGCAGTGTGGAGGGTGTTGTAT

* Primer used for extending and re-amplifying the potential recombination break points

Supplementary Table S2 The FPLV-specific primers used for the full-length genome sequencing of the FPLV NS gene. The primers were designed based on alignment of multiple FPLV genomes available in GenBank.

Primer name	Sequence (5'-3')
**FPV-NS-Fext**	GACCGTTACTGACATTCGCTTC
**FPV-NS-Rext**	GAAGGGTTAGTTGGTTCTCC
**FPV-NS-Fint**	GTTGAAACCACAGTGACGACAG
**FPV-NS-Rint**	CATCATCCARTCTTCAGGTG
**FPV-2161F**	TTGGCGTTACTCACAAAGACGTRC
**FPV-3475R**	GTTGGTGTGCCACTAGTTCCAGTA

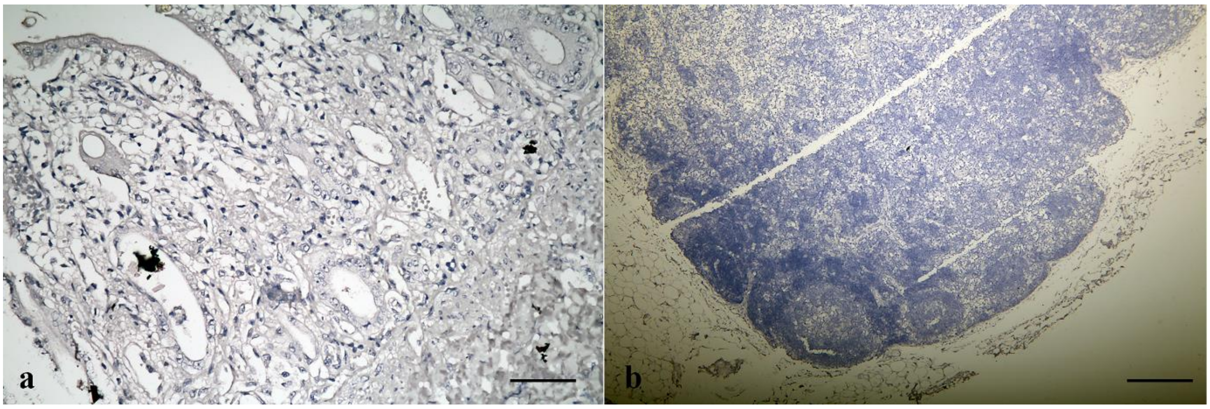

Supplementary Figure S1 Negative controls for FBoV-1 ISH. (a) Small intestine, cat no. 3. No reaction is present with an unrelated probe targeting feline herpesvirus incubated slide. Scale bar, 170 µm. (b) Submandibular lymph node, cat no.1. No signal is present with the unrelated probe incubated slide. Scale bar, 850 µm.
